# C-ITS Relevant Critical Vehicle-to-Vehicle Accident Scenarios for Accident Analysis

**DOI:** 10.3390/s22093562

**Published:** 2022-05-07

**Authors:** Maximilian Bauder, Klaus Böhm, Tibor Kubjatko, Lothar Wech, Hans-Georg Schweiger

**Affiliations:** 1 Center of Automotive Research on Integrated Safety Systems and Measurement Area, Technical University of Applied Sciences Ingolstadt, 85049 Ingolstadt, Germany; lothar.wech@thi.de (L.W.); hans-georg.schweiger@thi.de (H.-G.S.); 2 Department of Mechanical, Automotive and Aeronautical Engineering, Munich University of Applied Science, 80335 München, Germany; klaus.boehm@hm.edu; 3 Institute of Forensic Research and Education, University of Zilina, 010 26 Žilina, Slovakia; tibor.kubjatko@gmail.com

**Keywords:** cooperative intelligent transportation systems, C-ITS, V2X, accident scenarios, accident analysis, accident database

## Abstract

The relevance of scientific investigations, whether simulative or empirical, is strongly related to the environment used and the scenarios associated with it. Within the field of cooperative intelligent transport systems, use-cases are defined to describe the benefits of applications. This has already been conducted in the available safety-relevant Day 1 applications longitudinal and intersection collision risk warning through the respective technical specifications. However, the relevance of traffic scenarios is always a function of accident severity and frequency of a retrospective consideration of accident databases. In this study, vehicle-to-vehicle scenarios with high frequency and/or severe personal injuries are therefore determined with the help of the CISS database and linked to the use-cases of the safety-relevant Day 1 applications. The relevance of the scenarios thus results on the one hand from the classical parameters of retrospective accident analysis and on the other hand from the coverage by the named vehicle-to-x applications. As a result, accident scenarios with oncoming vehicles are the most relevant scenarios for investigations with cooperative intelligent transport systems. In addition, high coverage of the most critical scenarios within the use-cases of longitudinal and intersection collision risk warning is already apparent.

## 1. Introduction

Cooperative intelligent transport systems (C-ITS) are objects involved in traffic that exchange messages directly with each other to realise various applications [1]. If the objects are exclusively vehicles, it is known as vehicle-to-vehicle communication (V2V). If the vehicles exchange information with the infrastructure, it is known as vehicle-to-infrastructure communication (V2I). In general, a vehicle’s communication with its environment is referred to as vehicle-to-everything communication (V2X) [1,2]. Vehicles with V2X are already on the market today and have been built in large-scale production by VW since 2019 [3]. The currently available development stage is referred to as Day 1. This includes two applications, longitudinal and intersection collision risk warning (LCRW, ICRW), that send a warning to the driver in the event of a critical driving situation [4,5]. Due to the high complexity of traffic, use-cases were defined for a possible implementation of these applications within the respective technical specifications [6,7].

An investigation of the coverage of the specified use-cases to real accidents was carried out in 2011 by [8]. The study served to validate the coverage of the use-cases of the accident avoidance functions for vehicle-to-vehicle communication with critical accident scenarios, as the applications were still in the development phase at that time. The analysis was based on the American accident history between 2004 and 2008. In the article, 17 scenarios were identified, prioritised and grouped. Prioritisation was based on comprehensive economic cost and functional years lost, which is indirectly a function of accident frequency and severity. The scenarios found in this way were then compared with the five use-cases of the Vehicle Safety Communications-Applications (VSC-A) project developed at that time, but without reordering the relevance of the derived accident scenarios [8]. Since then, the relevance of the V2X use-cases concerning real accident scenarios has not been further addressed in the literature. Instead, the latest studies focus on scenarios for the validation of driver assistance systems and/or automated driving functions [9,10].

Therefore, this study aims to derive and prioritise safety-critical accident scenarios according to today’s accident occurrences and the now defined use-cases of the safety-relevant applications, LCRW and ICRW. This should lead to a study space for future investigations in the context of vehicle communication. In contrast to the 2011 study, the use-cases themselves are considered in addition to the criticality of the accidents and contribute to the relevance and prioritisation of the scenarios. The criticality of the vehicle-to-vehicle accident scenarios is also determined in direct relation to accidents involving personal injury, which either occur frequently or lead to serious personal injuries. The identification and prioritisation of C-ITS relevant accident scenarios under these aspects is a novelty within the literature and contributes to the investigation of accident scenarios by significantly reducing traffic injuries in the context of V2X communication. This will ensure that V2X is investigated under relevant and critical conditions for accident analysis and vehicle safety.

To determine the accident scenarios, the accident databases Crash Investigation Sampling System (CISS) of the NHTSA and the German accident statistics (Destatis) of the Fachserie 8 Reihe 7, both from 2019, are examined with personal injuries. The accident database of the NHTSA is used due to the high depth of information and free availability to be able to derive scenarios for further investigations that are as detailed as possible (e.g., for simulations, ground truth tests, etc.). Since the applications LCRW and ICRW were defined within Europe, the transferability of the accident statistics according to the CISS to the European area is investigated based on a small study. The German accident statistic is used as a representative of this.

After validating the transferability of the American accident occurrence to the German one, the accident occurrence is further concretised by considering the crash configuration and crash type of the CISS database. The findings are sorted and schematically described according to their absolute and relative frequency of occurrence as well as personal injuries. By aggregating the crash types within the CISS, it is possible to obtain an initial rough scenario description to enable assignment to the V2X use-cases. Afterwards, the use-cases of the safety-relevant collision risk applications LCRW and ICRW are assigned to the aggregated crash types and measured with a weighting factor. Two approaches for determining the weighting factors are followed and compared. The mean value of the sum of different weighting factors then gives the relevance of the scenario, which leads to the result of this work.

The use-cases of the safety-relevant applications LCRW and ICRW represent situations that lead to the triggering of a warning to the driver due to an imminent collision [6,7]. Thus, they define the application area of V2X Day 1 applications within vehicle safety. The use-cases of LCRW describe scenarios that occur in longitudinal traffic where the two vehicles involved are either driving in the same or opposite direction [7]. The use-cases of ICRW revolve around critical events in the area of intersections [6]. A list of all use-cases, as well as illustrations and descriptions, can be found in [6,7].

## 2. Materials and Methods

**Figure 1:**
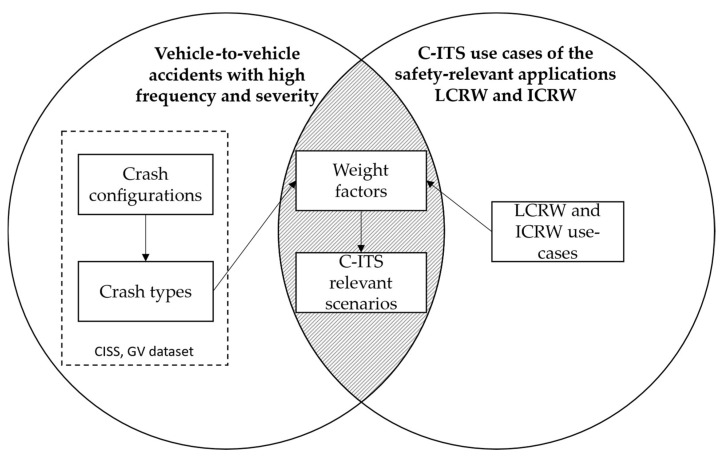
Schematic representation of the methodological approach for determining the C-ITS relevant scenarios.

In the following, the method for determining the C-ITS relevant accident scenarios is presented. Figure 1 schematically shows the approach followed. In principle, the intersection area of frequently occurring accidents or with severe personal injuries and the use-cases of the safety-relevant V2X applications LCRW and ICRW should be formed (shaded area). Scenarios located in the intersection area are critical from the point of view of road safety and within the effective range of current V2X applications. The scenarios in this intersection area thus represent an interesting area of investigation within vehicle safety in the context of vehicle communication. At the same time, “white spots” may be uncovered by identifying critical scenarios that are not yet covered by V2X use-cases. To achieve this, both the scenarios and the use-cases are weighted and compared, resulting in the order of critical scenarios. The scenarios are derived from the CISS database of crash configurations and types and are pre-sorted with regard to accident frequency and severity (see Section 2.2).

The weighting of the use-cases is then based on the theoretical effectiveness within the identified crash types. Two approaches are pursued, compared and discussed (see Section 3.2).

According to the European ETSI standardisation, it is assumed that both crash partners have an interoperable Day 1 communication architecture, that cooperative infrastructure is available, and that the use-cases are implemented according to the technical specifications [6,7].

### 2.1. Accident Databases and Transferability

The Crash Investigation Sampling System database of the National Highway Traffic Safety Administration (NHTSA) contains traffic accidents involving at least one passenger vehicle that had to be towed away from the accident scene. The sampling of accidents guarantees the representativeness of the overall accident occurrence. The NHTSA aims with the CISS database to provide a free and detailed database for researchers and developers to analyse motor vehicle accidents and resulting injuries. For each accident, up to 600 parameters are collected in 39 different data sheets [11,12,13]. The General Vehicle dataset from 2019, used in this study, summarises the most important parameters in one dataset. It contains 105 parameters (columns) with 4983 crash data samples (rows) [11,14].

The Fachserie 8 Reihe 7 contains all accidents recorded by the police in Germany that occurred in traffic on public roads or in public places [15]. Within kinds of accidents 1–5, which describe exclusively vehicle-to-vehicle accidents, 190,045 accidents were registered in 2019, which are used for the transferability analysis [15]. The 2019 datasets are used to avoid considering pandemic-related effects because of the COVID-19 pandemic occurring since 2020.

There are two difficulties to show the transferability of the accident data between CISS and Fachserie 8 Reihe 7, which are addressed in the following.

The first problem is the different classification of vehicle-to-vehicle accidents in the databases. Within the selected data of the Fachserie 8 Reihe 7, the accidents are classified according to the first five kinds of accidents, which describe vehicle-to-vehicle crashes and can be seen in Table 1 on the left side [15]. Since the CISS database does not use the same breakdown by kind of accident, the accident categories used here for crash classification are assigned on the right-hand side for transferability of the accident events. The table shows that a one-to-one assignment is not possible, except for accident type 4, which describes a collision between two oncoming vehicles and has an equivalent accident category within the CISS database (3: Same Trafficway, Opposite Direction) [16]. Due to this and the additional large difference in the number of accidents within the databases, only considering the relative distribution of crash severity is of interest in validating the transferability. Suppose the relative distribution of crash severity is of a similar order of magnitude between the accident types and crash categories. In that case, it can be assumed, at least to a certain extent, that the American accident occurrence is transferable to the German one. As this analysis is not the focus of this paper but is intended to strengthen the validity and informative value of the results, the transferability will not be examined in further detail.

The other difficulty is the comparability of accident severity. Within the German accident statistics, a distinction is made between those who are slightly injured, those who are seriously injured, and those who are dead [15]. Seriously injured persons are involved in an accident who are in stationary treatment for at least 24 h immediately after the accident [15]. Within the CISS database, on the other hand, the accident severity is specified according to the Maximum Abbreviated Injury Scale (MAIS) level (VAIS column) [17]. Since this study only differentiates between accidents with minor and severe personal injuries, including fatalities, a limit must be defined within the AIS level. In this evaluation, the findings of the European Commission from 2013 are followed, and serious injuries are described with a maximum AIS value of 3 or more (3+) [18].

### 2.2. Method to Determine C-ITS Relevant Scenarios

As shown in Figure 1, the GV dataset of the CISS database from 2019 is evaluated concerning crash configurations and crash types to determine the investigated scenarios. For the investigation of vehicle-to-vehicle crashes within the CISS, the crash configurations D to L are considered. To exclude crashes against a tree/pole, the parameter TREEPOLE is set to 0. This leads to 1273 accidents that are taken into account for this investigation. The method described below aims to identify scenarios according to the criteria mentioned (frequency and severity) and to describe the level of detail sufficiently precisely for the assignment of the use-cases. During the analysis, non-frequent or severe crash configurations or types are already discarded.

**Figure 2:**
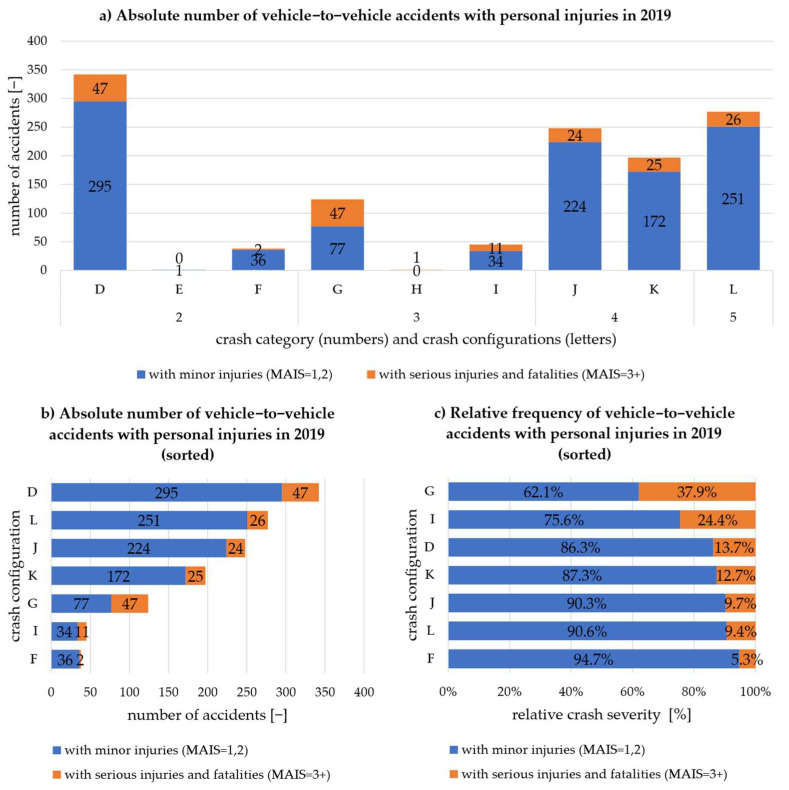
Vehicle-to-vehicle crashes with personal injury in 2019 according to CISS [19] broken down into crash configurations: (**a**) absolute number of all crash configurations; (**b**) absolute number of all relevant crash configurations sorted; (**c**) relative frequency of severe crashes sorted by the largest relative proportion of serious injuries and fatalities.

Figure 2 shows the breakdown of accidents with personal injury according to the crash configurations in the CISS database, which are briefly described in Table 2. Figure 2a shows the absolute number of accidents of all crash configurations of the considered crash categories. It can be seen that crash configurations E and H do not occur or only occur once and are therefore not considered further. With regard to the sorted absolute and relative frequency of vehicle-to-vehicle accidents, shown in (b) and (c), the rear-end crash is the most frequently occurring accident configuration. At the same time, the rear-end crash is the third most frequent (13.7%) to lead to serious accident consequences for the occupants. A higher relative frequency of severe accident consequences is only found in configurations G and I, representing accidents with oncoming vehicles. This is plausible due to the higher relative speeds. Fortunately, the absolute number of such accidents is among the lowest. Much more frequent are accidents in intersections with intersecting trajectories (L) or due to turning and lane-changing operations (J, K). However, the relative crash severity is lower than for the other configurations. The least critical accident situation is represented by two vehicles passing each other in the same direction (F), which can also be plausibly explained by crash mechanics (low-speed differences, much distance available for energy dissipation). For this reason, configuration F is also no longer considered for further investigation.

For the assignment of the use-cases is it necessary to consider the subdivision of the crash configurations into crash types. The crash types already describe the crash constellation of the accident in more detail. Crash type 20, for example, describes a vehicle that drives into the rear of another stationary vehicle, whereby a distinction is made between whether the vehicle in front continues to drive straight ahead (crash type 21) or wants to turn left or right (crash types 22 and 23) [16].

Since the use-cases describe scenarios with both vehicles involved in the accident, which in this case corresponds to a rear-end crash with a stationary front vehicle, the crash types that describe this scenario are aggregated into one crash type. In the above case, crash types 20 + 21 + 22 + 23 correspond to a rear-end collision with a stationary vehicle. This procedure was carried out for all crash types within the crash configurations. The result and a short description of the aggregated crash types, which in the following represent the scenario space for this investigation, can be found in Table 3.

**Figure 3:**
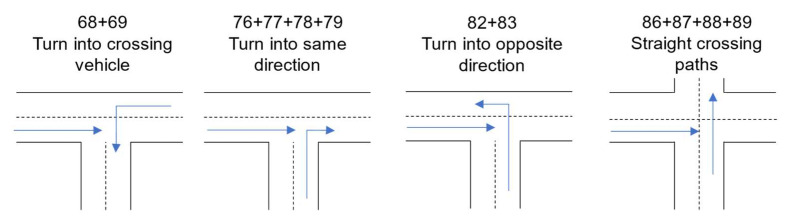
Schematic illustration of the crossing scenarios according to [16].

To better illustrate the differentiation of the crossing scenarios, they are shown schematically in Figure 3.

**Figure 4:**
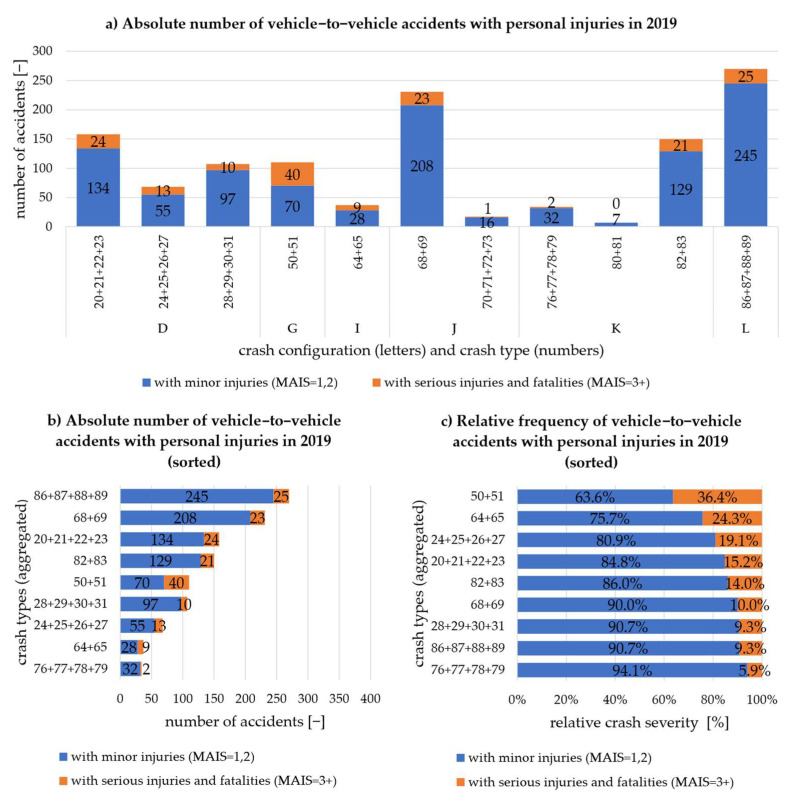
Vehicle-to-vehicle crashes with personal injury in 2019 according to CISS [19] broken down into crash types: (**a**) absolute number of all crash configurations; (**b**) absolute number of all relevant crash configurations sorted; (**c**) relative frequency of severe crashes sorted by the largest relative proportion of serious injuries and fatalities.

Figure 4a shows all crash types in an aggregated form of the crash configurations that will still be considered. As can be seen, there are crash configurations that can be further subdivided (D and K) and configurations that only describe one scenario (G).

Due to the low absolute number of cases, the aggregated crash types 70 + 71 + 72 + 73: ”Turn across path” and 80 + 81: “Turn into opposite directions” are not considered further.

As already described in Figure 2, Figure 4b shows the absolute number of vehicle-to-vehicle accidents of the respective crash types and (c) the relative frequency of the crash severity. Vehicles with intersecting trajectories in the intersection area occur most frequently but have the 2nd lowest relative crash severity. The greatest relative crash severity is again seen in vehicles crashing head-on (50 + 51), with a medium absolute number of occurrences. Despite the lower number of cases, severe personal injuries also occur most frequently in absolute terms, why this crash type (50 + 51) is to be assessed as the most critical. Crash type 76 + 77 + 78 + 79: “Turn into same direction” is the least critical in terms of absolute number and relative frequency of serious personal injuries.

The ranking of the scenarios in terms of accident frequency and severity cannot be derived directly from Figure 4b,c, as the ranking of the scenarios differs in between.

The ranking should therefore be determined on the basis of various weighting factors, which describe the relevance of the scenarios. For this purpose, three weighting factors (1–3) are introduced for accident frequency and severity.

(1) $$G_{1}=\frac{n_{crashes\;crash\;type}}{n_{all\;crashes}}$$

(2) $$G_{2}=\frac{n_{severe\;crashes\;within\;crash\;types}}{n_{all\;severe\;crashes}}$$

(3) $$G_{3}=\frac{n_{severe\;crashes\;within\;crash\;type}}{n_{all\;crashes\;within\;crash\;type}}$$

The factor $G_{1}$ is used to weight the accident frequency, which is formed from the quotient of the number of accidents of the respective scenario (crash type) and the number of all accidents. $G_{2}$ weights the number of severe accidents within the scenario against all severe accidents in the scenario area of investigation. Finally, $G_{3}$ weights the relative crash severity of the scenario itself. In terms of accident data, the relevance of the scenarios is then derived from the mean value of the weighting factors, which can be seen in Equation (4). This results in a maximum score of 100 and a minimum of 0 for relevance. The more points, the higher the relevance.

(4) $$Relevance_{CISS}=\left(\frac{G_{1}+G_{2}+G_{3}}{3}\right)\cdot 100\;$$

In order to additionally consider the use-cases of V2X communication within the relevance, a further weighting factor *G*_4_ is introduced (5).

(5) $$Relevance_{CISS+C-ITS}=\left(\frac{G_{1}+G_{2}+G_{3}+G_{4}}{4}\right)\cdot 100$$

Two approaches based on the same methodological procedure are used to determine $G_{4}$, which is described below. In principle, the applicability of the use-cases of LCRW and ICRW within the scenarios is considered to derive a weighting factor.

In the first approach, only use-cases that could be implemented through V2V communication only, without intelligent infrastructure are considered to determine the weighting factor. The use-cases of LCRW and ICRW that fulfil this condition are shown in Table 4. If a “yes” is noted in the column next to the respective use-case, it can be implemented without intelligent infrastructure and is relevant for the first approach.

Table 5 shows the assignment of the use-cases from Table 4 with the respective aggregated scenarios. The ticks symbolise the applicability of the scenario within the use-cases.

It can be seen that with the exception of scenario 24 + 25 + 26 + 27: “Rear-end slow”, all relevant scenarios are covered by at least one use-case. On the other hand, there is one use-case “stability problem” that cannot be assigned to any scenario because the use-case refers to the road conditions (e.g., wet), which is outside the scope of the scenarios. For this reason, this use-case is not considered for the calculation of the weighting factor. The weighting factor $G_{4}$ is then calculated by dividing the applicable use-cases for the scenario by all possible applicable scenarios. Most of the assignments are apparent, e.g., stationary vehicle to rear-end stopped. However, a few of them need a short explanation. “Rear-end decelerating” has been linked to “Emergency electronic brake light”, as this use-case becomes active in case of strong deceleration. Although the use-case is not necessarily triggered by every deceleration that has led to a rear-end crash, this use-case is most likely to become active in this scenario. For “Turn into the same direction”, the use-case “safety relevant and lane change” was also linked, as this scenario describes merging at an intersection and merging processes on multilane roads. The scenario “Straight crossing paths” was linked to the use-case “Turning collision risk warning”, as this use-case is active for intersecting trajectories in the turning processes of the intersection area. The results of this first approach are presented in Section 3.2.

In the second approach, the applicability of V2V and V2I use-cases is used to determine the weighting factor. Only the use-cases “Collision risk warning for vehicles with missing radio connectivity” in ICRW and “Roadworks”, “Stability problem”, as well as “Collision risk warning from third party” in LCRW are not taken into account, as they are too general to be linked to a scenario.

Table 6 shows the assignment of the ICRW use-cases to the scenarios that are within the scope (intersections). The same applies to Table 7, which assigns the LCRW use-cases to the corresponding scenarios. The scenarios that are not applicable in each case are not shown in the tables. This results in 11 use-cases from ICRW and LCRW, which are taken into account to determine the weighting factor.

A significant difference to the first approach can be seen in the intersection area in particular, as intelligent infrastructure is logically increasingly used there and additional use-cases arise here. The assignment to the respective use-cases can thus be easily derived from the schematic representation. Scenario 68 + 69: “Turn into crossing vehicle”, for example, cannot lead to a “Stop sign violation warning” or “Priority violation warning”. For scenario 82 + 83: “Turn into opposite direction”, on the other hand, all possible infrastructure warnings are imaginable.

Since all use-cases of the LCRW application could already be used in the first approach, Table 7 shows the same assignment as in Table 5. The weighting of scenario 76 + 77 + 78 + 79: “Turn into same direction” resulting from Table 7 is added with the weighting factor from Table 6, which is shown in the last row of the last column of Table 7. With the help of the two approaches for determining the weighting factors $G_{4}$ to consider the V2X communication, the relevance of the scenarios can be determined in the following.

## 3. Results

### 3.1. Transferability of Accident Data

**Figure 5:**
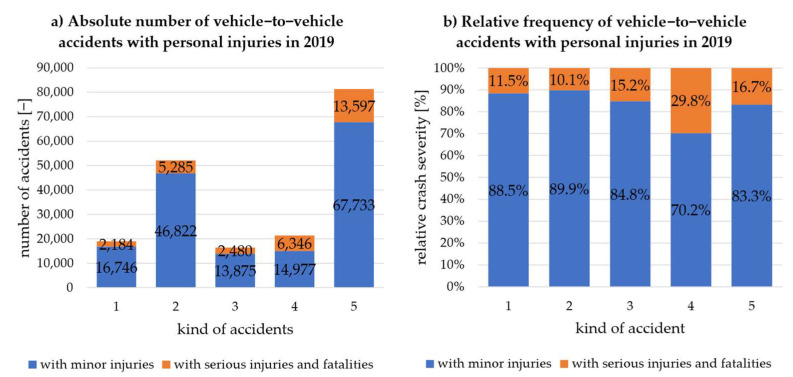
Vehicle-to-vehicle accidents with personal injury in 2019 according to Destatis [15]: (**a**) absolute number; (**b**) relative frequency.

**Figure 6:**
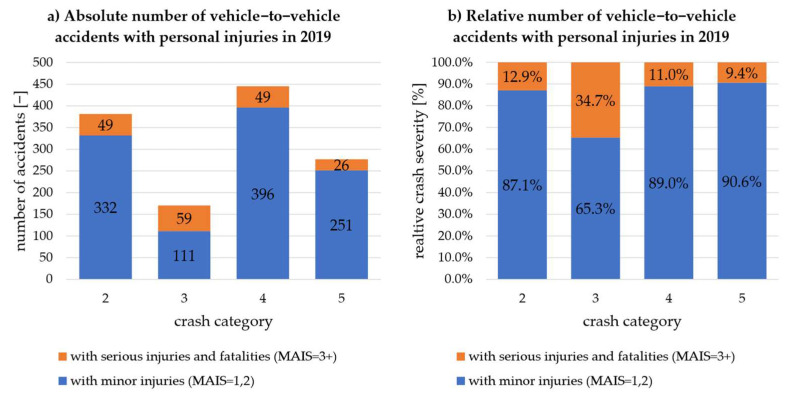
Vehicle-to-vehicle accidents with personal injuries in 2019 according to CISS [19]: (**a**) absolute number; (**b**) relative frequency.

According to the German statistics, the vehicle-to-vehicle accidents are shown in Figure 5 for the year 2019 across the five relevant kinds of accidents. On the left side (a), the absolute number of vehicle-to-vehicle accidents with personal injuries is plotted across the kind of accidents. The blue column represents the number of accidents with minor injuries, and the orange column with serious injuries and fatalities. The relative frequency of the crash severity resulting from (a) is shown on the right-hand side (b). Similarly, Figure 6 shows the accident occurrence according to the CISS database.

The unambiguous allocation of kind of accident 4 with crash category 3 shows that they have the highest relative proportion of serious injuries and fatalities in both cases. In addition, the relative distribution has a similar order of magnitude (about 1/3 of all accidents). This indicates an initial transferability of the accident occurrence within the CISS database for German accidents. When looking at the further percentage distributions of seriously injured and killed persons, a relative proportion of seriously injured and killed persons of 10% to almost 17% can be determined for all accident types or categories, despite ambiguous allocation. It can thus also be determined that the relative frequencies of accident consequences occur in a similar order of magnitude in both Germany and the USA. Therefore, the CISS database is considered valid for this investigation to determine C-ITS relevant scenarios that occur frequently and/or lead to serious personal injuries in the European area.

### 3.2. C-ITS Relevant Accident Scenarios

In the following, the relevance of the respective scenarios is calculated and compared without considering C-ITS and afterwards according to the two approaches described in Section 2.2. The calculation of the weighting factors $G_{1}$ to $G_{3}$ is shown below using scenario 50 + 51: “Head-on with lateral move” as an example.

According to Equation (1), the quotient results from 110 accidents within the investigated scenario divided by 1165 accidents in total, resulting in a value of $G_{1}\;$ = 0.094. To calculate $G_{2}$, the quotient is formed from the 40 serious injuries and fatalities within the scenario versus all 167 serious injuries and fatalities across all scenarios, resulting in a quotient value of $G_{2}\;$ = 0.240. Finally, $G_{3}$ is used to weight the relative crash severity within the scenario. This results in the quotient of 40 severe accidents by 110 accidents in total, leading to a value for $G_{3}$ of 0.364. According to Equation (4), without taking V2X into account, the value for $Relevance_{CISS}$ is 23.3.

**Figure 7:**
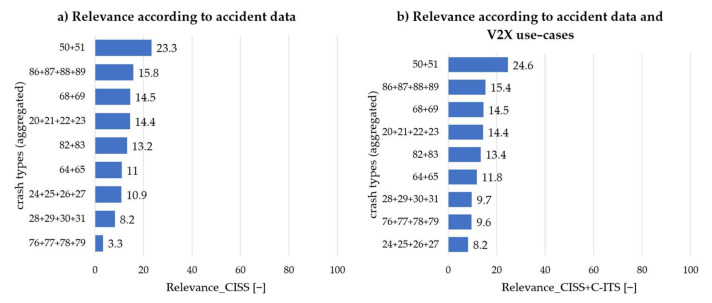
Sorting of the aggregated crash types according to importance (0 to 100): (**a**) without taking V2X into account; (**b**) with taking uses-cases of V2V only (approach 1) into account.

The result of all relevance calculations without V2X and according to approach 1, which only takes V2V communication into account, is shown in Figure 7. The relevance of the crash types without V2X is presented on the left. As expected, crash type 50 + 51: “Head-on with lateral move” is the most important. Crash type 76 + 77 + 78 + 79: “Turn into same direction” is consequently the most irrelevant scenario based on the accident data. This indicates that the approach chosen to determine relevance yields reasonable results. The twofold consideration of the crash severity also seems appropriate since the scenario that occurs most frequently in absolute terms (86 + 87 + 88 + 89: “Straight crossing paths”) follows in second place, and the weighting of the accident frequency can thus be assessed as sufficient. In general, it can be stated that there is a good mix in terms of relevance between scenarios in longitudinal traffic and the intersection area.

On the right side in Figure 7b the relevance according to approach one is shown. It can be seen here, that the order of the first six scenarios has not changed, which is also plausible since, according to Table 5, most scenarios were assigned to exactly one use-case. On the other hand, in the last three scenarios, there were some changes in place. Scenario 76 + 77 + 78 + 79: “Turn into same direction” was able to move in front of scenario 24 + 25 + 26 + 27: “Rear-end slow”, which could not be assigned to a use-case. Scenario 28 + 29 + 30 + 31: “Rear-end decelerating” was also able to move up one place.

Overall, according to approach 1, it can be seen that the ranking correlates strongly with the order according to the accident data and that the influence through V2V communication only plays a subordinate role. At the same time, it can also be stated that there is already a high coverage of the scenarios by the use-cases of the applications ICRW and LCRW.

**Figure 8:**
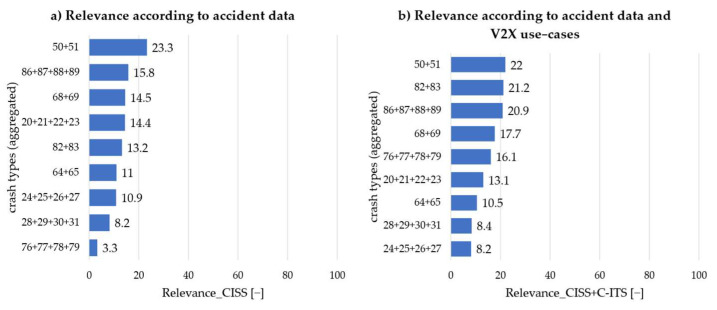
Sorting of the aggregated crash types according to importance (0 to 100): (**a**) without taking V2X into account; (**b**) with taking all relevant use-cases (approach 2) into account.

Finally, Figure 8 presents the change in relevance according to approach 2. On the left side, the relevance without V2X can be seen again. The right-hand side now shows larger shifts within the relevance. Although scenario 50 + 51: “Head-on with lateral move” is still the most relevant, the gap to second place has narrowed to 0.8 points. Scenarios in the intersection area exclusively occupy the following rankings. Scenario 82 + 83: “Turn into opposite direction” is just ahead of scenario 86 + 87 +88 + 89: “Straight crossing paths” This shows that V2X is expected to have a high impact in the intersection area now and in the future, which is mainly due to the use of intelligent infrastructure. Scenario 76 + 77 + 78 + 79: “Turn into same direction” represents the greatest improvement within relevance. This scenario climbed up to 5th place, starting from last place. Finally, rear-end scenarios are generally the most Irrelevant within the study area.

## 4. Discussion

Regarding the approaches to determine the relevance of V2X communication to the scenarios, it must be noted, that it is based only on the use-cases of the safety applications ICRW and LCRW. Existing further applications, such as Road Hazard Signaling (RHS), have not been considered in this study. This is because ICRW and LCRW become active directly before a critical event occurs and are, therefore, most relevant in relation to an accident.

Furthermore, it must be clarified that the described use-cases for determining the relevant scenarios do not represent a standard. The use-cases implemented by the vehicle manufacturers can therefore deviate from the use-cases considered in this study, which could also change the results shown here. Since the actual use-cases implemented by Volkswagen, for example, are not known, this could not be taken into account.

It should also be noted that the weighting of the use-cases of LCRW and ICRW according to Table 5, Table 6 and Table 7 was chosen according to the authors’ understanding of the description of the use-cases. The final result of this paper is, therefore, to a certain extent, dependent on the authors’ assessment and may change due to other assessments or approaches to determine the relevance of V2X to vehicle-to-vehicle accidents. The order of scenarios defined in this paper according to relevance should be further investigated and sharpened in future work.

## 5. Conclusions

The scope of this study was to create a relevant scenario space for future investigations in the field of vehicle communication. This was achieved by combining an evaluation of the American CISS database and the use-cases of the safety-relevant applications LCRW and ICRW to consider vehicle communication. Since these are European applications, the transferability of American accident events to European ones was first examined, using German accident events as a representative example.

The evaluation of the databases according to vehicle-to-vehicle accidents with personal injury shows that both the German and the American accident occurrences are in a comparable order of magnitude within the relative frequency of severe crashes. In this case, it can be considered valid to use the freely available CISS database of the NHTSA to get detailed crash information. The further subdivision of accidents down to the level of crash types within the CISS provides a good opportunity to compare accident scenarios with the use-cases of the safety-relevant applications LCRW and ICRW from C-ITS.

As a result, nine crash types could be identified as relevant scenario spaces, which led to frequent or severe accidents. By weighting the factors accident frequency, accident severity and coverage by V2X use-cases, the crash types with the highest relevance concerning these factors could be determined and correlated (sorted). Two approaches were followed, one considering only the use-cases of vehicle-to-vehicle communication and the other considering also intelligent infrastructure use-cases of ICRW and LCRW. The first approach shows that the influence of V2V communication on the relevance is small and is mainly generated by the accident data. Here, especially scenarios with oncoming vehicles are an important field of investigation. According to the first approach, the relevance of intersection scenarios and those in longitudinal traffic is also very balanced.

The results after the second approach show a significant increase in the relevance of scenarios in the intersection area, whereas frontal collisions remain the most important. This is justified by the additional usability of intelligent infrastructure within the intersection area. In general, the Day 1 applications investigated already show a high coverage of critical vehicle-to-vehicle accident scenarios.

Compared to the 2011 study, this work validated the coverage of the use-cases with the accident events and created a relevant scenario space for future investigations in the context of vehicle communication.

## Figures and Tables

**Table 1:** Linking of kinds of accidents according to [15] and crash categories according to [16,17].

Kinds of Accidents of Fachserie 8 Reihe 7 Collision with Another Vehicle Which…	Crash Categories of CISS Database
1. … starts driving, stops, or stands in stationary traffic	2: Same Trafficway, Same Direction 4: Changing Trafficway, Vehicle Turning
2. … is driving ahead or waiting	2: Same Trafficway, Same Direction
3. … is driving sideways in the same direction	2: Same Trafficway, Same Direction
4. … is oncoming	3: Same Trafficway, Opposite Direction
5. … is turning or crossing	4: Changing Trafficway, Vehicle Turning 5: Intersecting Paths

**Table 2:** Description of the crash configurations within the CISS database according to [16].

Configuration	Description
D: Rear-End	Rear-end crash of two vehicles travelling in the same direction.
E: Forward Impact	Rear-end crash after steering manoeuvre around a noninvolved object.
F: Sideswipe/Angle	Vehicles pass each other at a small angle when travelling in the same direction.
G: Head-On	Frontal crash of two oncoming vehicles.
H: Forward Impact	Frontal crash after steering manoeuvre around a noninvolved object.
I: Sideswipe/Angle	Vehicles pass each other sideways at a flat angle in the opposite direction of travel.
J: Turn Across Path	An accident resulting from a turning manoeuvre or lane change where one vehicle pulls in front of the other, both travelling in the same direction.
K: Turn Into Path	An accident resulting from a turning manoeuvre in which one vehicle pulls in front of the other, while they are travelling in opposite directions.
L: Straight Paths	An accident due to straight crossing trajectories.

**Table 3:** Description of the aggregated crash types within the CISS database according to [16].

Crash Types (Aggregated)	Description
20 + 21 + 22 + 23 Rear-end stopped	Vehicle drives into the rear of a stationary vehicle.
24 + 25 + 26 + 27 Rear-end slow	Vehicle drives into the rear of another slower moving vehicle.
28 + 29 + 30 + 31 Rear-end decelerating	Vehicle drives into the rear of a braking vehicle.
50 + 51 Head-on with lateral move	Frontal collision of two oncoming vehicles due to one vehicle leaving its lane of travel.
64 + 65 Sideswipe with lateral move	Sliding along of two oncoming vehicles due to one vehicle leaving the lane of travel.
68 + 69 Turn into crossing vehicle	Vehicle crosses the lane of an oncoming vehicle.
76 + 77 + 78 + 79 Turn into same direction	Vehicle turns right or left into the same lane as the other vehicle.
82 + 83 Turn into opposite direction	Left-turning vehicle intersects the trajectory of a vehicle travelling straight ahead from the left.
86 + 87 + 88 + 89 Straight crossing paths	Vehicles with straight trajectories collide laterally on the right or left at an intersection.

**Table 4:** Applicability of all use-cases of ICRW and LCRW according to [6,7] using vehicle-to-vehicle communication only.

Use-Cases *ICRW* and LCRW	Applicable with V2V
*Turning collision risk warning*	yes
*Merging collision risk warning*	yes
*Collision risk warning for vehicles with missing radio connectivity*	no
*Stop sign violation warning*	no
*Priority violation warning*	no
*Traffic light violation warning*	no
*Turning regulation warning*	no
Safety relevant lane change	yes
Emergency electronic brake light/traffic condition	yes
Roadworks	no
Stationary vehicle	yes
Stability problem	yes
Wrong-way vehicle driving	yes
Safety relevant vehicle overtaking	yes
Collision risk warning from third party	no

**Table 5:** Applicability of the V2V use-cases of *ICRW* and LCRW to the scenarios under investigation and calculation of the weighting factor G4.

Crash Types (Aggregated)/Use-Cases *ICRW* and LCRW	*Turning Collision Risk Warning*	*Merging Collision Risk Warning*	Safety Relevant Lane Change	Emergency Electronic Brake Light/Traffic Condition	Stationary Vehicle	Stability Problem	Wrong-Way Vehicle Driving	Safety Relevant Vehicle Overtaking	$\mathrm{Weighting}\;\mathrm{Factor}\;G_{4}$
20 + 21 + 22 + 23: Rear-end stopped					✓				1/7
24 + 25 + 26 + 27: Rear-end slow									0/7
28 + 29 + 30+ 31: Rear-end decelerating				✓					1/7
50 + 51: Head-on with lateral move							✓	✓	2/7
64 + 65: Sideswipe with lateral move								✓	1/7
68 + 69: Turn into crossing vehicle	✓								1/7
76 + 77 + 78 + 79: Turn into same direction		✓	✓						2/7
82 + 83: Turn into opposite direction	✓								1/7
86 + 87 + 88 + 89: Straight crossing paths	✓								1/7

**Table 6:** Applicability of V2V + V2I use-cases of *ICRW* to the scenarios under investigation and calculation of the weighting factor G4.

Crash Types (Aggregated)/Use-Cases *ICRW*	*Turning Collision Risk Warning*	*Merging Collision Risk Warning*	*Stop Sign Violation Warning*	*Priority Violation Warning*	*Traffic Light Violation Warning*	*Turning Regulation Warning*	$\mathrm{Weighting}\;\mathrm{Factor}\;G_{4}$
68 + 69: Turn into crossing vehicle	✓				✓	✓	3/11
76 + 77 + 78 + 79: Turn into same direction		✓	✓	✓	✓	✓	5/11
82 + 83: Turn into opposite direction	✓		✓	✓	✓	✓	5/11
86 + 87 + 88 + 89: Straight crossing paths	✓		✓	✓	✓		4/11

**Table 7:** Applicability of V2V + V2I use-cases of LCRW to the scenarios under investigation and calculation of the weighting factor G4.

Crash Types (Aggregated)/Use-Cases LCRW	Safety Relevant Lane Change	Emergency Electronic Brake Light/Traffic Condition	Stationary Vehicle	Wrong-Way Vehicle Driving	Safety Relevant Vehicle Overtaking	$\mathrm{Weighting}\;\mathrm{Factor}\;G_{4}$
20 + 21 + 22 + 23: Rear-end stopped			✓			1/11
24 + 25 + 26 + 27: Rear-end slow						0/11
28 + 29 + 30 + 31: Rear-end decelerating		✓				1/11
50 + 51: Head-on with lateral move				✓	✓	2/11
64 + 65: Sideswipe with lateral move					✓	1/11
76 + 77 + 78 + 79: Turn into same direction	✓					1/11 + 5/11 = 6/11

## Data Availability

Publicly available datasets were analyzed in this study. These data can be found here: https://www.nhtsa.gov/file-downloads?p=nhtsa/downloads/CISS/2019/ (accessed on 10 January 2022) and https://www.google.com/url?sa=t&rct=j&q=&esrc=s&source=web&cd=&cad=rja&uact=8&ved=2ahUKEwi6ucqrjrv2AhXPQvEDHcxSC6AQFnoECAkQAQ&url=https%3A%2F%2Fwww.destatis.de%2FDE%2FThemen%2FGesellschaft-Umwelt%2FVerkehrsunfaelle%2FPublikationen%2FDownloads-Verkehrsunfaelle%2Fverkehrsunfaelle-jahr-2080700197004.pdf%3F__blob%3DpublicationFile&usg=AOvVaw1oy6qKDdKpXNx1mUKPicB7 (accessed on 10 January 2022).
